# Understanding the differential impacts of two antidepressants on locomotion of freshwater snails (*Lymnaea stagnalis*)

**DOI:** 10.1007/s11356-024-31914-0

**Published:** 2024-01-17

**Authors:** Nandini Vasantha Raman, Asmita Dubey, Ellen van Donk, Eric von Elert, Miquel Lürling, Tânia V. Fernandes, Lisette N. de Senerpont Domis

**Affiliations:** 1 Department of Aquatic Ecology, Netherlands Institute of Ecology (NIOO-KNAW), Droevendaalsesteeg 10, 6708 PB Wageningen, The Netherlands; 2 Department of Aquatic Ecology and Water Quality Management, Wageningen University & Research, P.O. Box 47, 6708 PB Wageningen, The Netherlands; 3 Department of Environmental Biology, University of Utrecht, Utrecht, The Netherlands; 4 Aquatic Chemical Ecology, Biocenter, Institute of Zoology, University of Cologne, Cologne, Germany; 5 Department of Pervasive Systems, EEMCS, University of Twente & Department of Water Resources, ITC, University of Twente, Enschede, The Netherlands

**Keywords:** Psychopharmaceutical, SSRI, SNRI, Behavioral toxicity, Gastropod, Fluoxetine, Venlafaxine, 5α-cyprinol sulfate

## Abstract

There is growing evidence of negative impacts of antidepressants on behavior of aquatic non-target organisms. Accurate environmental risk assessment requires an understanding of whether antidepressants with similar modes of action have consistent negative impacts. Here, we tested the effect of acute exposure to two antidepressants, fluoxetine and venlafaxine (0–50 µg/L), on the behavior of non-target organism, i.e., freshwater pond snail, *Lymnaea stagnalis*. As compounds interact with chemical cues in the aquatic ecosystems, we also tested whether the effects altered in the presence of bile extract containing 5α-cyprinol sulfate (5α-CPS), a characterized kairomone of a natural predator, common carp (*Cyprinus carpio*). Behavior was studied using automated tracking and analysis of various locomotion parameters of *L. stagnalis.* Our results suggest that there are differences in the effects on locomotion upon exposure to venlafaxine and fluoxetine. We found strong evidence for a non-monotonic dose response on venlafaxine exposure, whereas fluoxetine only showed weak evidence of altered locomotion for a specific concentration. Combined exposure to compounds and 5α-CPS reduced the intensity of effects observed in the absence of 5α-CPS, possibly due to reduced bioavailability of the compounds. The results highlight the need for acknowledging different mechanisms of action among antidepressants while investigating their environmental risks. In addition, our results underline the importance of reporting non-significant effects and acknowledging individual variation in behavior for environmental risk assessment.

**Graphical Abstract:**

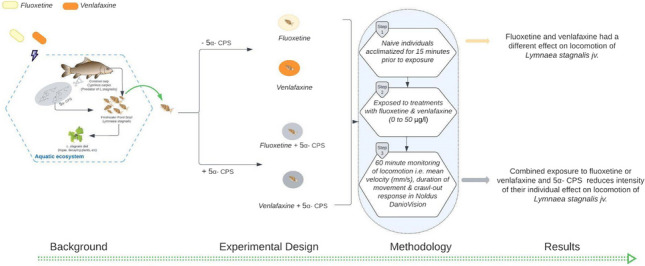

**Supplementary Information:**

The online version contains supplementary material available at 10.1007/s11356-024-31914-0.

## Introduction

Since industrialization, increasing amounts of synthetic chemicals are being discharged to the aquatic environment (Bernhardt et al. 2017). Pharmaceuticals are a group of synthetic chemicals that are contaminating aquatic ecosystems and hence included in the Water Framework Directive’s Watchlist of contaminants of emerging concern (Gomez Cortes et al. 2020). Pharmaceuticals are used to treat, prevent and/or diagnose a medical condition in humans and animals. Antidepressants are a major class of psychiatric pharmaceuticals that are increasingly occurring in aquatic systems in recent years (Sehonova et al. 2018; Silva et al. 2015). Global average fluoxetine (selective serotonin reuptake inhibitor) concentrations in surface waters ranges between 0.012 and 1.4 µg/L (Weinberger & Klaper 2014), while its concentration in wastewater treatment plant effluents was found to reach 3.5 µg/L (Salgado et al. 2011). Observed concentrations of venlafaxine (selective serotonin-norepinephrine reuptake inhibitor) in surface waters are variable ranging from 0.021 to 0.64 µg/L (Chen et al. 2022; de Jongh et al. 2012; Fernandes et al. 2020) with concentrations as high as 2.190 µg/L found in wastewater treatment plant effluent (Schultz and Furlong 2008).

Antidepressants can be classified into nine categories based on their mechanism of action, out of which selective serotonin reuptake inhibitors (SSRIs), tricyclic antidepressants, and serotonin-noradrenaline reuptake inhibitors (SNRIs) are widely used (Melchor-Martínez et al. 2021). SSRIs and SNRIs are two major categories of antidepressants that frequently occurring in aquatic systems. SSRIs increase neuronal activity by preventing presynaptic reuptake of serotonin (5-HT) at all administered concentrations. In contrast, SNRIs are another major category of antidepressants that have a dual mechanism of action, i.e., at low concentrations SNRIs inhibit reuptake of 5-HT similar to SSRIs, and when administered at high concentrations, they prevent the reuptake of both 5-HT and norepinephrine (NE) (Harvey et al. 2000; Salahinejad et al. 2022). In determining standard ecotoxicological metrics, these subtle differences in the mechanism of action among antidepressant categories are overlooked. Considering that the active receptors for antidepressants have been conserved in evolution among invertebrates and vertebrates (Tierney 2018), antidepressants may evoke a response in non-target organisms (Gómez-Canela et al. 2023). Hence, it is crucial to explore the direct and indirect effects of different categories of antidepressants on non-target organisms. It is also necessary to ascertain whether antidepressants with different mechanisms of actions lead to similar or different responses in organisms, which would determine whether there is a need to monitor antidepressants individually in contrast to grouping them under one umbrella group.

Two antidepressants, fluoxetine, an SSRI, and venlafaxine, an SNRI, were chosen for this study to test for sublethal direct and indirect effects. These two antidepressants are specifically of interest considering the recent discussions at European water authorities about the grouping of pharmaceuticals with similar mode of action for monitoring purposes. Venlafaxine and fluoxetine both inhibit the reuptake of neurotransmitter—serotonin—at low concentrations, but at high concentrations, venlafaxine switches to also inhibiting the reuptake of norepinephrine (Harvey et al. 2000). We selected these two pharmaceuticals because of their frequent occurrence in aquatic systems, similarity in mode of action, and lack of understanding about their potential to evoke direct and indirect effects on non-target organisms and to disrupt natural chemical compounds in aquatic ecosystems. For example, environmentally observed and sublethal concentrations of fluoxetine at 0.01 µg/L and 0.1 µg/L reduced the percentage of time spent on locomotion to < 20% in *Gammarus pulex* (De Lange et al. 2006).

In addition to synthetic chemicals, aquatic systems also have a smellscape of natural chemicals. Natural chemicals are chemicals that are released by either organisms or the environment through natural processes such as excretion, weathering, and decomposition (Klaschka 2009). A specific class of natural chemicals is represented by infochemicals, i.e., chemicals that are released by organisms and affect interactions such as recognizing kin, recognizing predators, choosing mates, and locating food (Van Donk et al. 2016). Different classes of chemical cues govern the interaction between prey and predator. Kairomones are such a cue released by the predator that is adaptively favorable for the prey, by alerting the prey of the presence of the predator. Other types of cues include disturbance cues released by startled prey and alarm cues released by injured prey (chemicals leaked by damaged tissue of prey) (Ferrari et al. 2010). Emerging synthetic chemicals such as pharmaceuticals have the potential to indirectly disrupt interaction between aquatic organisms (Van Donk et al. 2016). For example, antidepressants may affect the response of non-target organisms to infochemicals, such as fish kairomones (Bedrossiantz et al. 2021; Bellot et al. 2022). Despite these indirect effects being potentially equal in magnitude as the direct effects, they are currently not investigated in the standard ecotoxicological metrics (Preisser et al. 2005). As explained in Van Donk et al. (2016), indirect effects of synthetic chemicals on aquatic organisms can be density-mediated, trait-mediated, or via mimicking/disrupting infochemicals.

Freshwater snails are prey for other aquatic organisms such as fishes and crayfish and play a role in decomposition, making them a key organism in aquatic food webs. Therefore, it is important to inspect the direct and indirect effects of environmentally relevant concentrations of pharmaceuticals on this non-target group of organisms. The pulmonate freshwater snail, *Lymnaea stagnalis*, is a widespread gastropod that plays an important role in aquatic food webs as herbivores, occasionally feeding on small dead invertebrates, including congeners (Amorim et al. 2019; Ducrot et al. 2014). *L. stagnalis* is a sensitive ecosystem health indicator making this species a reliable model species in ecotoxicity tests to define standard ecotoxicological parameters such as no observed effect concentration (NOEC), lethal and effective concentrations of toxicants (Amorim et al. 2019; Fodor et al. 2020). The effect of toxicants on the behavior of *L. stagnalis* is sensitive and informative, specifically considering that behavioral traits such as avoidance and locomotion are highly energy consuming in *L. stagnalis* (Amorim et al. 2019). Common forms of locomotion in *L. stagnalis* such as crawling are regulated by serotonergic pathway by releasing serotonin to the muscle and cilia in the sole (Aonuma et al. 2020). Therefore, antidepressants that act on serotonin such as SSRIs and SNRIs have the potential to affect locomotion. To exemplify, Fong et al. (2015) observed that venlafaxine (157 µg/L to 3.13 mg/L concentrations) increased crawling speed in 2 marine snail species (i.e., *Urosalpinx cinerea* and *Lithopoma americanum*). In the same study, however, fluoxetine at high concentrations (3.45 mg/L) reduced the crawling speed of both snail species (Fong et al. 2015). Additionally, in the presence of infochemical cues from their natural predators, tench (*Tinca tinca*) and crayfish (*Procambrius clarkia*), *L. stagnalis* display an innate anti-predator response by crawling out of the water surface (crawl-out response) (Dalesman et al. 2006; Orr and Lukowiak 2010). *L. stagnalis* possesses serotonin receptors that are binding sites for both chosen antidepressants, hence indicating their potential to indirectly affect traits such as locomotion and/or interfere in the anti-predator response caused by the predator infochemical. Additionally, *L. stagnalis* possess receptors for octopamine, which are involved in locomotion. Octopamine is structurally similar to norepinephrine, i.e., a neurotransmitter regulated by venlafaxine at high concentrations (Bymaster et al. 2001). Differences in the direct and indirect effects of fluoxetine and venlafaxine on behavior of *L. stagnalis* at environmentally relevant concentrations and their potential to affect natural chemical information transfer remain unexplored.

In this study, therefore, we tested differences in the effect of these two representatives and widely used antidepressants (i.e., fluoxetine and venlafaxine) on behavior of freshwater snail, *Lymnaea stagnalis*. As we were interested in how the presence of a potential infochemical affected the impact of antidepressant exposure, we also assessed if effects caused by two antidepressants were modulated by the presence of a potential infochemical (carp bile extract with active kairomone (5α-CPS); Hahn et al. 2019). Several studies have shown behavioral responses such as locomotory responses to be rapid and instinctive; therefore, we focused on the response of individuals upon acute exposure (Bossus et al. 2014; De Lange et al. 2006; Ford and Fong 2016). We hypothesized the response pattern along the exposure gradient varies between fluoxetine and venlafaxine, owing to the differences in the receptors they act upon (Fong et al. 2015). More specifically, due to this different mode of actions, we expected exposure to both fluoxetine and venlafaxine individually to increase locomotion in *L. stagnalis* seen as increasing velocity (mm/s) and duration of movement (s) and crawl-out over the concentration gradient. Additionally, we hypothesized that the presence of a kairomone from a natural predator (carp bile extract with active kairomone(5α-CPS) would modulate the differences in the effects caused by venlafaxine and fluoxetine on the locomotion of *L. stagnalis*, i.e., mean velocity (mm/s), duration of movement (s) and proportion of crawl-out.

## Methodology

We tested the effect of two widely used antidepressants fluoxetine and venlafaxine on behavior of freshwater snail, *Lymnaea stagnalis*, using a randomized block design, to account for the effect of timing (experimental day) on the experimental results. Each block represents an experimental day, and the experiments were done over the same time period, with 96 snails for each antidepressant (*n* = 192) randomly assigned to one block. To reflect the environmental relevant concentrations that affected locomotion in *Gammarus pulex* (De Lange et al. 2006), we exposed juveniles of *L. stagnalis* to a replicated concentration gradient of antidepressants (fluoxetine or venlafaxine) ranging from 0 to 50 µg/L (0, 0.01, 0.05, 0.1, 0.5, 1, 10, 50 µg/L) in six replicates. To test the blocking effect in our experimental design, we carried out 36 additional trials with control treatments where *L. stagnalis* juveniles were exposed to aerated groundwater. The snails were exposed to each of the antidepressant concentrations separately and in combination with a fixed concentration of carp bile extract with active kairomone (5α-cyprinol sulfate, 5α-CPS; Hahn et al. 2019). The behavior of the acclimatized snails was tracked in 6-well plates (volume: 16.8 mL; each well representing an experimental unit) for a period of 60 min using the Noldus Daniovision system (Noldus, The Netherlands). The tracking was done in the presence and absence of carp bile extract with active kairomone (5α-CPS) to test potential of fluoxetine and venlafaxine to disrupt the anti-predator response. We also performed a degradation experiment to account for photodegradation of both chosen antidepressants over the course of the experiment.

### Collection and maintenance of snails

*Lymnaea stagnalis* individuals were collected from the ponds located at the Netherlands Institute of Ecology (NIOO-KNAW), Wageningen, The Netherlands (51° 59′ 17.3″ N 5° 40′ 25.0″ E). Twelve adult snails were transferred to plastic tanks containing 20 L of aerated groundwater and cultured under a light regime of 16:8 h at room temperature (20 °C). The snails were fed butterhead lettuce ad libitum (Large et al. 2006). To maintain good water quality and remove organic waste, the water was completely replaced with clean groundwater every week, until the adult snails had laid eggs along the surface of the tank. Subsequently, half of the water was replaced in order to reduce the loss of eggs and hatchlings. Juvenile snails of similar size (aged 2 to 7 days post-hatching) were used for behavior-tracking experiments considering their high sensitivity to chemical pollutants and higher locomotory activity in comparison to the adults (Gérard et al. 2005; Mazur et al. 2016). A batch of juveniles were fed and then collected from the rearing tanks by filtering the water from tanks with 110-µm sieve and added to a clean tank of aerated groundwater 24 h prior to the bioassay to keep them free from the alarm signals of conspecifics.

### Kairomone and pharmaceuticals

Kairomone used in the study was carp bile extract containing 5 alpha-cyprinol sulfate (5α- CPS). The bile was extracted using the method described in Hahn et al. (2019). In short, the bile was extracted by C18-solid-phase and eluted with pure methanol to a volume of 1 mL. The bile salt was quantified by an UHPLC system with an Accela 1250 psi pump coupled with an Exactive Orbitrap mass spectrometer (Thermo Fisher). This extract was determined to contain 30 ng/µL of 5α-CPS. Two hundred microliters of the bile was diluted in 500 mL of aged, aerated groundwater (referred to as groundwater medium) which resulted in a 12 ng/mL final concentration of 5α-CPS.

Fluoxetine hydrochloride, N-methyl-3-phenyl-3-[4-(trifluoromethyl) phenoxy] propan-1-amine; hydrochloride (CAS 56296–78-7), a serotonin reuptake inhibitor; venlafaxine hydrochloride, 1-[2-(dimethylamino)-1-(4-methoxyphenyl) ethyl] cyclohexan-1-ol (CAS 99300–78-4), serotonin and norepinephrine reuptake inhibitor were purchased from Merck, Sigma-Aldrich. One milligram per liter stock of both fluoxetine and venlafaxine was prepared from which dilutions of 100 µg/L and 10 µg/L were prepared using MilliQ water. Stocks were prepared daily to avoid potential loss of compounds due to degradation which would otherwise lead to variations in the final concentrations.

### Behavioral tracking

As a first step to behavioral tracking, naive *L. stagnalis* individuals were acclimatized in the 6-well plate (one individual per well) with 7.5 mL of groundwater medium for 15 min (Well’s plate Company: Falcon; dimensions: 12 cm × 8 cm × 2 cm; material: polystyrene; well volume: 16.8 mL). Subsequently, all the wells were spiked with pharmaceutical using stock solutions (i.e., 1 mg/L stock for exposure concentrations 10 and 50 µg/L; 100 µg/L stock for 1 µg/L; 10 µg/L stock for < 1 µg/L), and groundwater as control. Finally, the total volume at required concentrations was made up to 15 mL with only groundwater or groundwater containing 5α-CPS kairomone. The individuals were exposed to a certain concentration from a concentration gradient of fluoxetine or venlafaxine ranging between 0.01 and 50 µg/L, while the concentration of 5α-CPS was kept at 12 ng/mL (1.87 µM) which is approximately ninefold higher than the 107 pM concentration that induced behavioral responses in *Daphnia magna* (Hahn et al. 2019). The Noldus Daniovision system with the EthoVision software was used for the behavioral tracking (Noldus et al. 2001; Valenti et al. 2012). The software setup consists of experimental settings, arena settings, trial control settings, and detection settings. Experimental settings were set to live tracking, and the videos were recorded using Basler GeniCam (Basler acA1300-60) with a frame rate of 60 fps and resolution of 1280 × 1024. The six arenas representing each well on the 6-well plates were placed in the DanioVision apparatus, the wells were automatically detected as arenas in the arena settings, and the arenas were calibrated at 80 mm according to the size of the plate. Trial control settings were set to track for 1 h with 1 min of acclimation time, and snails in the wells were recognized using auto-detect settings in DanioVision setup. All the trials were carried out during the light period (10:00 till 18:00 in July, i.e., summer period in The Netherlands), and 48 trials were run per day. All the experimental units were tracked for behavioral parameters such as mean velocity (mm/s), and cumulative duration of movement(s) in DanioVision tracking system with 60 s preparation time and 3600 s, i.e., 60 min tracking. The tracking time of 60 min was based on the understanding that behavioral changes to contaminant exposure are instinctive and immediate and yet ecologically significant considering that behavioral changes are energy consuming and alter interactions with predator, prey, and conspecifics (Weis et al. 2001). The tracks were recorded, and videos were used to visually determine which snails crawled out of the arena and the time point of the experiment at which they crawled out.

### Validation of experimental design

#### Chemical analysis

To validate the exact exposure concentration of fluoxetine and venlafaxine, concentrations in water from each well were quantified using LC–MS/MS(Agilent 1290 Infinity II liquid chromatography-triple quadrupole (LC-QQQ, Agilent). We took samples at the end of the trial. Samples (500 µL) were collected at the exposure concentrations < 1 µg/L and diluted with 70% acetonitrile (v/v in MilliQ) to a final volume of 1 mL and stored at 4 °C until quantification. The sample volume was reduced to 300 µL in concentrations > 1 µg/L to avoid matrix effects such as carryover affecting the accuracy of quantification. Diluted samples were quantified using LC–MS/MS in ESI ( +) mode with Zorbax SB-C18 column (1.8 µm) for separation. Both the compounds were detected in a multiple reaction monitoring in positive mode with transitions m/z 310.1—> 148.2 for fluoxetine and m/z 278.2—> 121.2 for venlafaxine. The aliquots needed to establish a calibration curve of fluoxetine and venlafaxine were prepared in MilliQ water with physical properties similar to groundwater. The concentration of the stock solutions was confirmed and for quantification of concentrations in the experimental units, the limit of quantification was determined (for details, see Supplement 1,1).

Additionally, all the diluted samples stored with 70% acetonitrile (v/v in MilliQ) were qualitatively analyzed for the presence of kairomone, 5α-CPS in the carp bile extract used for the exposure. Zorbax SB-C18 column (1.8 µm) was used for separation, and the presence of 5α- CPS in the samples was confirmed by the abundant m/z fraction 531.29986 [M–H]^–^ in ESI (-) mode (Hahn et al. 2019).

The quantitative analysis software of Masshunter (QQQ, Agilent) was used to quantify the concentration of fluoxetine and venlafaxine as well as to confirm the presence of 5α-CPS in samples containing the kairomone. The bile used in the experiment was used as the standard against which the presence/ absence of 5α-CPS was confirmed in the samples from experimental treatments.

#### Degradation experiment

Pharmaceutical degradation under environmental conditions is inevitable. This can be through various degradation pathways such as photodegradation, biodegradation, hydrolysis, photolysis, and oxidation (Andrés-Costa et al. 2017; Kwon and Armbrust 2006; Rúa-Gómez and Püttmann 2013; Sterr and Sommaruga 2008). In order to account for the potential loss of the antidepressants during our exposure experiments, we determined the fate of both venlafaxine and fluoxetine under our experimental conditions. Triplicates of 15 mL of aerated groundwater with fluoxetine hydrochloride and venlafaxine concentrations 0, 1, 10, and 100 µg/L were incubated at 20 °C with continuous light. From each experimental unit < 100 µg/L, 500 µL of samples were collected at 5 sampling timepoints: initially and after, 1, 8, 24, and 48 h. Aliquots of 100 µL were collected from the units exposed to 100 µg/L to avoid carryover effects caused by higher concentrations hindering quantification. All collected samples were stored in amber vials with the volume brought up to 1 mL using 70% acetonitrile (v/v in MilliQ) for further analyses. Diluted samples with fluoxetine and venlafaxine in 70% acetonitrile (v/v in MilliQ) were quantified by LC–MS/MS (see “Data analysis”).

Fluoxetine in our experimental conditions followed first-order kinetics; therefore, we applied *mkin* package (Ranke et al. 2023) in Rstudio (version 4.0.3) to determine the dissipation time-50 (DT_50_), i.e., the time required for 50% of the initial concentration of fluoxetine or venlafaxine to dissipate from our experimental system.

### Data analysis

Raw data output from EthovisionXT was used to calculate mean velocity (mm/s), and cumulative duration of movement (s) for 60 min. The data was quality controlled by visually inspecting each recording, with removal of unrealistic values caused by the snail crawling out, immobility affecting detection or detection issues due to low visibility caused by the snail being in the edges of the wells plate. The outlier values were removed based on the inter quartile range method for outlier detection for each treatment (Boratyński et al. 2012).

In order to test for a blocking effect in our experimental design, we carried out 36 additional trials with control treatments where naive *L. stagnalis* juveniles, i.e., offspring from the pool of 6 parents were exposed to aerated groundwater in the absence of fluoxetine, venlafaxine, and kairomone. As the aim was to test for potential blocking effects of the day of experiment, we spread the additional testing over 6 days, similar to the experimental runs. The behavioral changes in *L. stagnalis* juveniles were tracked following the same steps as the trials of experimental treatments using the Daniovision apparatus. As we detected a blocking effect (see for details Supplement 1.3), we proceeded with analyzing trait-mediated indirect effects of fluoxetine and venlafaxine on locomotion parameters using log response ratios. Log response ratio (LRR) is an effect size ratio determined as a natural logarithm of the ratio of a response variable (in this case, velocity (mm/s) and duration of movement (s) in an experimental treatment to that of a control treatment) (Hedges et al. 1999). Further statistical analyses were performed using R (version 4.0.3).

Tests were performed using linear mixed effects models (lme), using the *lmer* function in the “lme4” package (Bates et al. 2023). Two lme tests were performed: first to compare the effect of fluoxetine and venlafaxine on mean velocity and cumulative duration of movement, and second, to test the effect of fluoxetine/venlafaxine in the absence of kairomone in comparison to fluoxetine/venlafaxine in the presence of kairomone, on mean velocity and cumulative duration of movement. Logistic regression was used to test crawl-out frequency on exposure to the two compounds in the absence/presence of kairomone. Lastly, we calculated Cohen’s *d* using the method from Brysbaert and Stevens (2018) to evaluate the strength of our statistical claims. Details on these calculations can be found in Supplement 1.4.

The residuals of LRR of endpoints, i.e., mean velocity and total duration, were then checked for normality (Shapiro–Wilk test) and homogeneity of variance (Levene’s test) (Shapiro and Wilk 1965).The effect of compound exposure on the LRRs was tested using submodels with day as the random factor and fixed effects namely only concentration (model 1), only compound (model 2), and combined effect of concentration and compound (model 3). These submodels were tested against a zero model (effect on LRRs in the absence of both concentration and compound in fixed effects). Using the model with the combined effect of concentration and compound, pairwise comparisons were made within and between the compounds.

In order to test if the natural chemical compound modulates the effects caused by the two antidepressants, we also tested the effect of carp bile extract with the natural chemical compound, 5α-CPS on the locomotion parameters, i.e., time taken to crawl-out of the water surface, mean velocity and cumulative duration of movement in comparison to control. To test the effects of simultaneous exposure to kairomone and compound, a model with compound (fluoxetine or venlafaxine), concentration, and kairomone (absent/present) was set as fixed effects and day of the experiment as the random factor. Pairwise comparisons were performed using the “emmeans’’ package (Lenth et al. 2023). We used the language of evidence (Muff et al. 2022) to report and interpret the outcome of our statistical tests. To this end, *p*-values have been interpreted according to the approximate range within which the *p*-value falls (range 0.1–1 indicating little or no evidence, 0.05–0.1 as weak evidence, 0.01–0.05 as moderate evidence, 0.001–0.01 as strong evidence, and < 0.001 as very strong evidence).

## Results

### Validation of experimental design

#### Chemical analysis

We observed that measured concentration of fluoxetine and venlafaxine in our experimental matrix (i.e., groundwater) was unaffected by the presence of kairomone carp bile extract. The average measured concentrations of fluoxetine in experimental treatments (both in the presence and absence of kairomone) containing 0.5, 1, 10, and 50 µg/L were 0.14 (s.d. ± 0.08), 0.19 (s.d. ± 0.17), 6.92 (s.d. ± 0.4), and 37.1 (s.d. ± 5.74) µg/L, respectively. Average measured concentrations in treatments with expected venlafaxine concentrations of 10 and 50 µg/L (both in the presence and absence of kairomone) were 2.09 (s.d. ± 0.12) and 25.08 (s.d. ± 8. 58) µg/L. 5α-CPS was qualitatively confirmed to be present in all the samples containing bile extract. The degradation pattern of the two compounds in our experimental conditions was also measured for validation of the setup (Supplement 1.2).

### Differences in trait-mediated effects of venlafaxine and fluoxetine

**Fig. 1:**
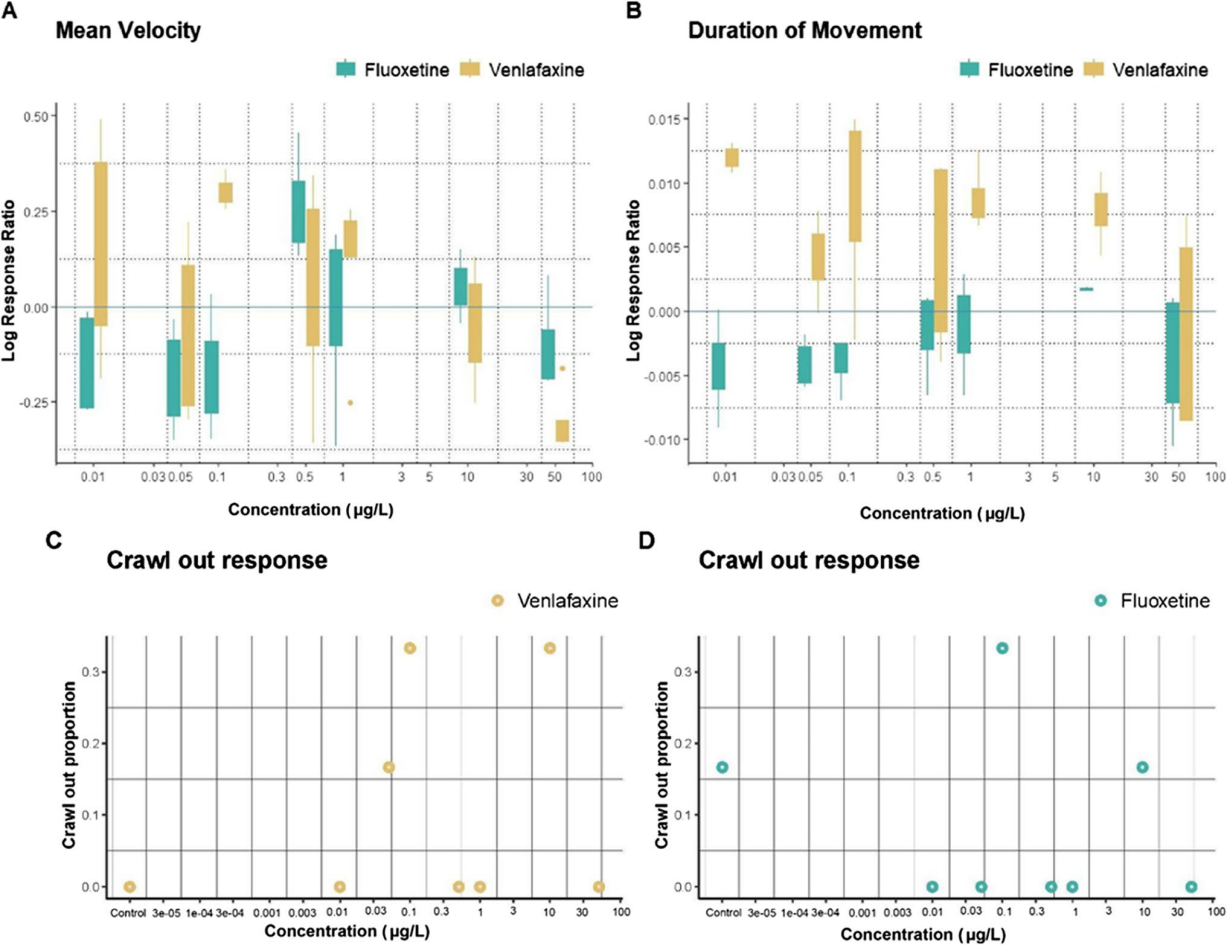
Trait-mediated effects of fluoxetine (green) and venlafaxine (yellow) concentrations (0, 0.01, 0.05, 0.1, 0.5, 1, 10, 50 µg/L) on locomotion of *L. stagnalis*. **A** Mean velocity: Log response ratio of average mean velocity of venlafaxine (yellow boxes) and fluoxetine (green boxes) with no-effect (i.e., 0 LRR) represented by the blue solid line. **B** Duration of movement: Log response ratio of cumulative duration of movement of venlafaxine (yellow boxes) and fluoxetine (green boxes), with no-effect (i.e., 0 LRR) represented by blue solid line; Positive log response ratio indicates an increase; negative indicates a decrease in mean velocity/ duration of movement. The whiskers in the box plot represent the interquartile range (IQR) and extend to the minimum and maximum values 1.5 times the IQR. Yellow and green whiskers indicate IQR in venlafaxine and fluoxetine treatment respectively. **C** Venlafaxine crawl-out response: Proportion of the total number of replicates that crawled out when exposed to venlafaxine (yellow dots) over a concentration gradient. **D** Fluoxetine crawl-out response: Proportion of the total number of replicates that crawled out when exposed to fluoxetine (green dots) over a concentration gradient

Log response ratio (LRR) of velocity and duration of movement of an experimental treatment relative to the control (Fig. 1A, B) exhibit a difference in response for venlafaxine and fluoxetine. Based on the lme model (combined effect of concentration and compound), pairwise comparisons were made within and between the two compounds. Within the compound (along the concentration gradient), venlafaxine was observed to show a non-monotonic (biphasic) dose response (NMDR) with stimulation at lower concentrations and inhibition at the highest concentration. Fluoxetine did show a different pattern in response and had values closer to the control line, except for one concentration (Fig. 1A). Comparisons between the two compounds revealed that at lower concentrations 0.01 μg/L, 0.1 μg/L, 1 μg/L, exposure to venlafaxine caused an increase in cumulative duration of movement relative to the control in contrast to exposure to fluoxetine which led to a decrease in cumulative duration of movement relative to the control (Fig. 1B). The results have been detailed below using the language of evidence (Muff et al. 2022).

#### Main effects and interaction effects

Regardless of the compound, concentration had a moderate effect on mean velocity (*P* = 0.03) and cumulative duration of movement (*P* = 0.03) (Model 1). The type of compound, i.e., venlafaxine vs. fluoxetine, had no effect on mean velocity and a very strong effect on cumulative duration of movement (*P* < 0.001) (Model 2). A combined effect of compound and concentration showed a very strong effect on mean velocity (*P* < 0.001) and cumulative duration of movement (*P* < 0.001) (Model 3).

#### Pairwise comparisons within the concentration gradient for each compound

Pairwise comparisons were made from the lme model with a combined effect of concentration and compound. The lowest concentration of venlafaxine 0.01 μg/L showed moderate to strong evidence of an increase in cumulative duration of movement when compared to concentrations of venlafaxine 0.05 µg/L (*P* = 0.02), 0.5 µg/L (*P* = 0.03), 50 µg/L (*P* < 0.001) (Fig. 1B). However, the highest concentration of venlafaxine 50 µg/L showed moderate to strong evidence of decrease in mean velocity when compared to lower concentrations 0.01 µg/L(*P* = 0.03), 0.1 µg/L (*P* = 0.008), 0.5 µg/L (*P* = 0.08), 1 µg/L (*P* = 0.03) (Fig. 1A). Similarly for cumulative duration of movement, the highest concentration of venlafaxine 50 µg/L showed moderate to very strong decrease when compared to the lower concentrations 0.01 µg/L (*P* < 0.001), 0.1 µg/L (*P* = 0.03), 0.5 µg/L (*P* = 0.09), 1 µg/L (*P* = 0.01), 10 µg/L (*P* = 0.02) (Fig. 1B). Pairwise comparisons of fluoxetine exhibited weak evidence of an increase in mean velocity for concentration 0.5 µg/L when compared to 0.01 µg/L (*P* = 0.06), 0.05 µg/L (*P* = 0.03), 0.1 µg/L (*P* = 0.06), 50 µg/L(P = 0.07); no effects were observed for cumulative duration of movement (Fig. 1A, B).

#### Pairwise comparisons between the two compounds

Using a lme model with a combined effect of concentration and compound, pairwise comparisons were also made for the same concentration between the two compounds (e.g., response in LRRs for venlafaxine 0.01 µg/L vs fluoxetine 0.01 µg/L). There was moderate evidence of difference in mean velocity between the two compounds for concentration 0.1 µg/L (*P* = 0.05). Cumulative duration of movement showed moderate evidence of difference between the two compounds for the concentrations 0.01 µg/L (*P* = 0.02), 0.1 µg/L (*P* = 0.04), 1 µg/L (*P* = 0.04), with an increase in cumulative duration of movement for venlafaxine in comparison to a slight decrease observed for fluoxetine (Fig. 1B).

#### Crawl-out response

Additionally, we determined the differences in the effect of fluoxetine and venlafaxine on crawl-out by comparing the crawl-out proportion (i.e., number of animals crawled out out of the total number of animals (*n* = 6)) as seen in Fig. 1C and D. Overall, we did not record a 100% crawl-out along the concentration gradient for both fluoxetine and venlafaxine exposure. However, 2 of 6 individuals (i.e., 0.33 proportion) crawled out when exposed to venlafaxine at 0.5 µg/L, 0.1 µg/L, and 10 µg/L concentrations. Contrastingly, no crawl-out was observed upon 0.5 µg/L fluoxetine exposure. The results from logistic regression analysis show no significant results for both fluoxetine and venlafaxine throughout the exposure gradient.

### Effect of fish kairomone on locomotion of *L. stagnalis*

**Fig. 2:**
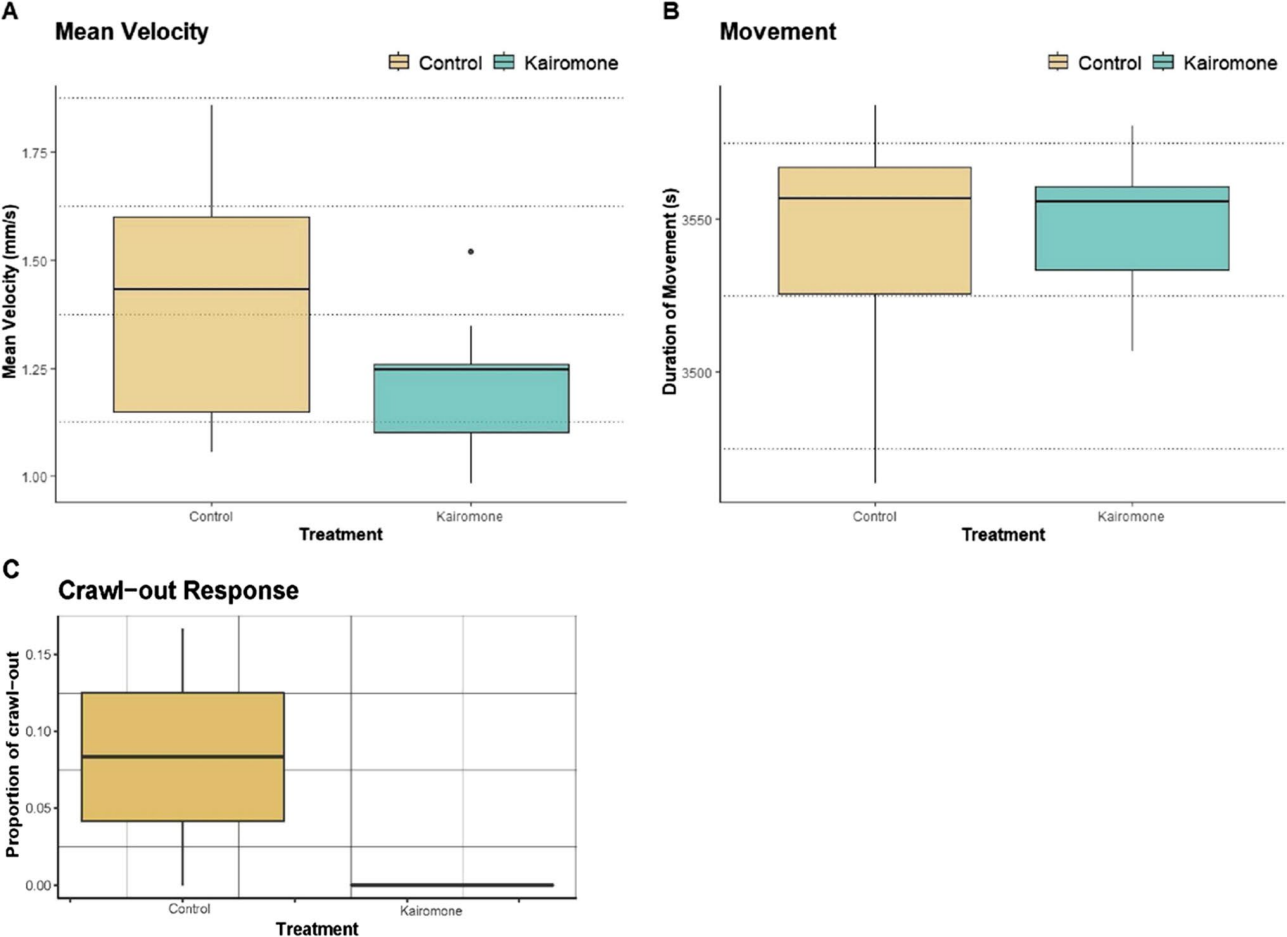
Effect of carp bile extract with natural chemical compound, kairomone 5α-CPS on the locomotion of *L.stagnalis* in comparison to non-antidepressant, non-5α-CPS exposed controls. **A** Mean velocity: Average mean velocity (mm/s) of control (yellow boxes) and kairomone, 5α-CPS (green boxes). **B** Duration of movement: Cumulative duration of movement (s) of control (yellow boxes) and kairomone, 5α-CPS (green boxes). **C** Crawl-out response: Proportion of the total number of replicates that crawled out when exposed to control (yellow boxes) and kairomone, 5α-CPS (green boxes). The whiskers in the box plot represent the interquartile range (IQR) and extend to the minimum and maximum values 1.5 times IQR. Yellow and green whiskers indicate IQR in control and kairomone, 5α-CPS treatment respectively

As a first step in testing the effects of fluoxetine and venlafaxine on the locomotion of *L. stagnalis* in the presence of fish kairomones, we tested the effects of solely the fish kairomone (bile extract with 5α-CPS) on the locomotion of *L. stagnalis.* The effect of carp bile extract with 5α-CPS was evaluated by comparing the mean velocity (mm/s), cumulative duration of movement (s), and proportion of crawl-out of only 5α-CPS exposed treatments with no 5α-CPS, non-pharmaceutical exposed controls (Fig. 2). There was no evidence of the effect of the bile extract when compared to the control for mean velocity (*P* = 0.34), cumulative duration of movement (*P* = 0.67), and crawl-out response (*P* = 0.9).

### Effects of venlafaxine and fluoxetine in the presence of fish kairomone

**Fig. 3:**
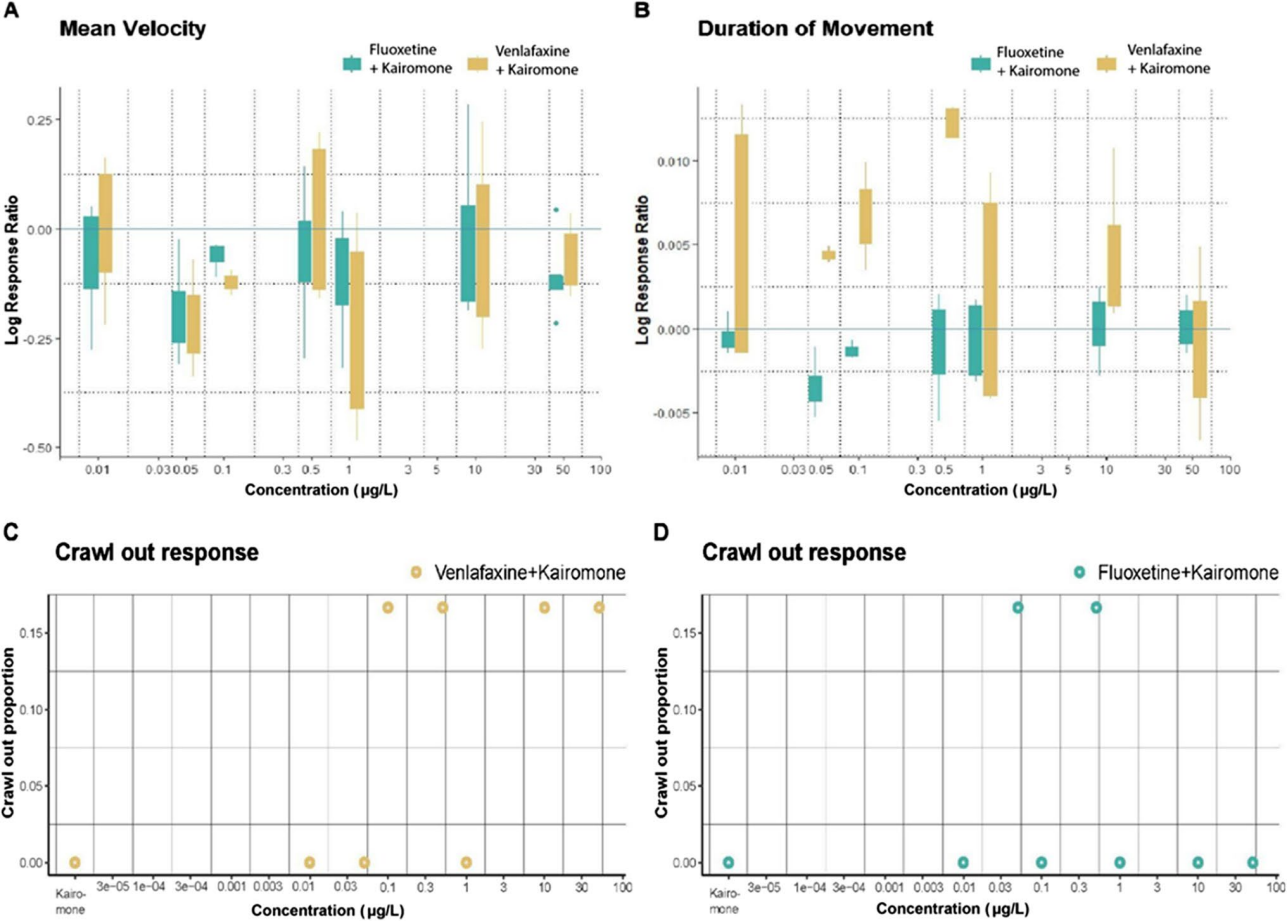
Effect of fluoxetine (green) and venlafaxine (yellow) concentrations (0, 0.01, 0.05, 0.1, 0.5, 1, 10, 50 µg/L) on locomotion of *L.stagnalis* in the presence of 5α-CPS (kairomone). **A** Mean velocity: Log response ratio of average mean velocity of venlafaxine (yellow boxes) and fluoxetine (green boxes) in the presence of 5α-CPS with no-effect represented by the blue solid line. **B** Duration of movement: Log response ratio of cumulative duration of movement of venlafaxine (yellow boxes) and fluoxetine (green boxes) in the presence of 5α-CPS with no-effect represented by blue solid line; Positive log response ratio indicates an increase, negative indicates a decrease in mean velocity/ duration of movement. The whiskers in the box plot represent the interquartile range (IQR) and extend to the minimum and maximum values 1.5 times IQR. Yellow and green whiskers indicate IQR in venlafaxine + kairomone and fluoxetine + kairomone treatment respectively. **C** Venlafaxine + 5α-CPS crawl-out response: Proportion of the total number of replicates that crawled out when exposed to venlafaxine in the presence of 5α-CPS (yellow dots) over a concentration gradient. **D** Fluoxetine + 5α-CPS crawl-out response: Proportion of the total number of replicates that crawled out when exposed to fluoxetine in the presence of 5α-CPS (green dots) over a concentration gradient

To test the differences in treatments on simultaneous exposure to pharmaceutical and kairomone, a model with combined effects of compound (fluoxetine or venlafaxine), kairomone(5α-CPS) (absent or present), and concentration was used (Fig. 3A, B). The results observed in the presence of kairomone were found to be similar to the results in the absence of kairomone as mentioned above, but the effects were weaker with higher *p*-values. Although similar to crawl-out response in the absence of kairomone, logistic regression analysis showed a lack of significant effect on crawl-out when exposed simultaneously to venlafaxine and 5α-CPS (Fig. 3C) or to fluoxetine and 5α-CPS (Fig. 3D).

#### Pairwise comparisons within the concentration gradient for each compound in the presence of fish kairomone

In the presence of the fish kairomone, venlafaxine showed a NMDR response only for cumulative duration of movement although there was weaker evidence in comparison to the moderate to strong evidence in the absence of 5α-CPS (the “Pairwise comparisons within the concentration gradient for each compound” section). Additionally, in the presence of 5α-CPS, there was evidence of an increase in the cumulative duration of movement on exposure to 0.5 µg/L of venlafaxine, which was not observed in the absence of 5α-CPS (Figs. 1B, 3B). There were weak effects observed on mean velocity on exposure to fluoxetine in the absence of 5α-CPS; however, there was no evidence of effects on locomotion parameters on exposure to fluoxetine in the presence of 5α-CPS (Figs. 1A, 3A). The lowest concentration of venlafaxine 0.01 µg/L showed moderate evidence of an increase in cumulative duration in comparison to the highest concentration 50 µg/L (*P* = 0.03). There was moderate to very strong evidence of an increase in cumulative duration of movement for concentration 0.5 µg/L in comparison to 0.05 µg/L (*P* = 0.04), 1 µg/L (*P* = 0.007), 10 µg/L (*P* = 0.03), 50 µg/L (*P* < 0.001) (Fig. 3B). Pairwise comparisons with the concentration gradient for fluoxetine showed no effects for either of the locomotion parameters in the presence of 5α-CPS (Fig. 3A, B).

#### Pairwise comparisons between the two compounds in the presence of fish kairomone

Pairwise comparison between the compounds (e.g., response in LRRs for venlafaxine 0.01 µg/L in the presence of 5α-CPS vs fluoxetine 0.01 µg/L in the presence of 5α-CPS) showed weak evidence of difference for low concentrations 0.01 µg/L (*P* = 0.09), 0.05 µg/L (*P* = 0.08) and very strong evidence for 0.5 µg/L (*P* < 0.001) (Fig. 3B), in comparison to the moderate evidence observed in the absence of 5α-CPS (“Pairwise comparisons between the two compounds” section), except for venlafaxine 0.5 µg/L where there is a strong increase in cumulative duration of movement in the presence of 5α-CPS. Pairwise comparisons for the same concentration between the two compounds showed no evidence of difference for mean velocity (Fig. 3A).

## Discussion

Antidepressants alter the receptors serotonin and/or norepinephrine both of which are evolutionarily conserved and control locomotion in molluscs. Recently, the adaptive role of serotonin in animal behavior and cognition both in invertebrates and vertebrates has been highlighted (Bacqué-Cazenave et al. 2020). In this study, we examined the effects of two antidepressants on the non-target organism *Lymnaea stagnalis*, considering environmentally relevant concentrations and their impact on energy-consuming behaviors such as locomotion and anti-predator responses alongside natural chemical compound, i.e., a kairomone 5α-CPS.

### Differences in trait-mediated effects of venlafaxine and fluoxetine

Our results show that venlafaxine and fluoxetine vary in their effects on the behavior of *L.stagnalis*. For venlafaxine, we observed a non-monotonic response with an increase in cumulative duration of movement at the lower concentrations and decrease in both locomotion parameters at the highest concentration. Exposure to fluoxetine did not alter locomotion except for weak evidence of an increase of mean velocity for concentration 0.5 µg/L. On comparing LRRs between the two compounds, effects on locomotion in lower and environmentally relevant concentrations (0.01 µg/L, 0.1 µg/L, 1 µg/L) were also found to be significantly different between fluoxetine and venlafaxine. The cumulative duration of movement increased when exposed to venlafaxine, whereas it decreased when exposed to fluoxetine.

Differences in response to venlafaxine and fluoxetine exposure have also been observed in other studies. Fong et al. (2015) found clear effects of venlafaxine with an increased crawling speed of two marine snails, i.e., *Lithopoma* and *Urosalpinx*, while fluoxetine reduced crawling speed. In our study, we observed a NMDR for venlafaxine and no strong effects of fluoxetine on locomotion. The discrepancy between the Fong et al. (2015) study and ours could be because our study was focused on the lower concentration gradient between 0.01 and 50 µg/L, while Fong et al. (2015) used higher concentrations ranging from 3.13 µg/L to 3.13 mg/L. In addition, exposure duration was longer in the study of Fong et al. (2015) compared to our study, 4 h vs 1 h, respectively. Furthermore, Fong et al. (2015) applied a “before and after” design because of the considerable between-animal variation in crawling speed. Both studies confirm a variation in response between the antidepressants. This can be due to the differences in their mechanism of action, i.e., along with the serotonin reuptake inhibition similar to fluoxetine; venlafaxine also inhibits the reuptake of norepinephrine (also known as noradrenaline- a neurotransmitter). Similar to serotonin, octopamine, another neurotransmitter involved in regulating locomotion in gastropods, is structurally similar to norepinephrine and has affinity to norepinephrine receptors (Gerhardt et al. 1997). Therefore, venlafaxine has the potential influence to locomotion in *L. stagnalis* via serotonin and norepinephrine receptors. The alteration in locomotion observed at ecologically relevant concentrations of venlafaxine in our study has ecological implications such as increased risk of predation and modifications in crawling out behavior (Fong et al. 2015). Changes in behavior can be considered an early warning signal for more serious ecological implications (Hellou 2011).

The high variability in trait-mediated effects observed in this study highlights that antidepressants with similar modes of action such as SSRI and SNRIs should not be grouped together in environmental risk assessments. Compounds within the class of SSRI antidepressants themselves have been shown to have high variability in chemical structures (Coleman and Gouaux 2018), which can lead to differences in response upon exposure. Differences in activity, feeding, and chemotaxis of *Caenorhabditis elegans* in responses to 2 SSRIs (sertraline and fluoxetine) were reported by van der Most et al. (2023). Instead of group- monitoring based on therapeutic groups, grouping pharmaceutical compounds based on chemical structure similarities can be a potential alternative (Davey et al. 2022).

Additionally, trait-mediated effects of venlafaxine followed a non-monotonic dose response (NMDR), i.e., stimulation at low concentrations and inhibition at high concentrations in our study (see the “Differences in trait-mediated effects of venlafaxine and fluoxetine” section). Non-monotonic dose responses are commonly reported in toxicity studies with antidepressants (Bossus et al. 2014; Fong et al. 2017; Rivetti et al. 2016; van der Most et al. 2023). In our study, we did not observe a NMDR relationship for the trait-mediated effects of fluoxetine on *L.stagnalis*. This is contrary to the observations of other studies where such a relationship over low concentrations of fluoxetine has been detected (Fong et al. 2017; Guler and Ford 2010; Painter et al. 2009). Despite the increasing evidence on non-monotonic responses, the mechanisms behind these antidepressants induced non-monotonic responses remain unexplored. However, several potential mechanisms for NMDR include protective and beneficial effects at low doses that reduce or disappear at high concentrations have been theorized as summarized by van der Most et al. (2023) such as (i) receptor desensitization or limited receptor availability; (ii) alternative mechanisms of action; (iii) multiple receptors with different affinities to the antidepressant; (iv) high dose acute toxicity caused by saturation or overt toxicity modulating the endpoint being measured (also see, Hill et al., (2018).

In this study, fluoxetine did not have a significant effect except for one concentration. In addition, both the compounds did not evoke an anti-predator response, i.e., crawling out of the water surface. The absence of detectable effects on locomotion has also been reported in juvenile cuttlefish exposed to fluoxetine (Di Poi et al. 2014). It is necessary to report such non-significant results, considering the existing citation bias in ecotoxicology where studies with significant positive or negative effects are more cited than the studies with no significant effect (Hanson et al. 2018). It is also important to consider the contribution of power analysis of the sample size (see supplement 1.4). In order to achieve precision in the environmental risk assessment of these antidepressants, it is crucial to expand the horizon and look beyond the significant effects these compounds have on aquatic organisms.

### Effect of venlafaxine and fluoxetine in the presence of kairomone

In nature, synthetic chemicals such as antidepressants are exposed to a range of naturally occurring compounds in aquatic ecosystems (Van Donk et al. 2016); hence, it is interesting to study the effects of antidepressant exposure in the presence of naturally occurring compounds such as kairomones. Previous studies have studied anti-predator behavior in *L.stagnalis* with tench (*Tinca tinca*), causing crawl-out response in the presence of predator and alarm cues (Dalesman et al. 2006). In this study, we wanted to test the effect of predator cues from *Cyprinus carpio*, a cyprinid like *T.tinca* containing CPS in bile*.* Hahn et al. (2019) observed that 5α-CPS obtained from *C.carpio* bile induced diel vertical migration in *Daphnia magna*, which is a predator avoidance behavior. We aimed to study whether the presence of 5α-CPS would also induce anti-predator response in *L. stagnalis* and if its presence alters the response of *L. stagnalis* to the antidepressants. The results show the 5α-CPS itself did not induce crawling response or alter locomotion parameters. Exposure to antidepressants in the presence of 5α-CPS reduced the intensity of effects seen in the absence of 5α-CPS. This was observed for both venlafaxine and fluoxetine. For our study, we used bile extract which contained 5α-CPS. A possible explanation is that the bile salts could have interacted with the antidepressants and reduced the bioavailability of the compounds for *Lymnaea stagnalis.* While the quantification of antidepressant concentrations provides information about their presence or absence which was not affected by the presence of kairomone in our study, it does not necessarily capture all the mechanisms by which they can influence organisms. Behavioral responses can be influenced by various factors, including neurochemical interactions, receptor binding, and downstream signaling pathways, which may affect the responses of *L.stagnalis* to antidepressants in the presence of kairomone-5α-CPS.

### Fate of venlafaxine and fluoxetine in our experiment

Our study focused on an extensive concentration gradient of fluoxetine and venlafaxine, i.e., 0.01, to 50 µg/L following their environmentally measured concentrations. Due to analytical limitations, lower exposure concentrations in our behavioral tracking experiment were not quantifiable by LC/MS–MS. To overcome this limitation and confirm the presence of fluoxetine and venlafaxine in our behavioral tracking experiments, we determined the half-life of fluoxetine and venlafaxine by performing a separate follow-up experiment dedicated to determining the fate of these compounds in our experimental conditions. In our degradation experiment, we found negligible photodegradation of both fluoxetine and venlafaxine in our experimental matrix under similar light and temperature conditions confirming a negligible loss of fluoxetine and venlafaxine over the 8 h of behavioral tracking that a stock was being used. The half-life of both compounds determined for an initial concentration 100 µg/L is higher than both our exposure duration (i.e., 60 min) and 8 h (i.e., total number of trials run using the same stock solution). These determined half-lives of fluoxetine and venlafaxine suggest that all the responses observed are neither influenced by loss of the active compound or increased degradation product. Kwon and Armbrust (2006) reported that fluoxetine is persistent to hydrolysis and photolysis with a half-life over 100 days. In another study, Yin et al. (2017) observed half-lives of fluoxetine and venlafaxine to be 46.6–183.2 days and 68.8–145.4 days respectively upon photodegradation.

However, in our behavioral tracking, the nominal concentrations of fluoxetine were as low as 15% at low concentrations and around 74% at highest concentrations, while the measured concentrations of venlafaxine were 20% and 50% of the nominal concentrations indicating a considerable loss of these compounds. It is important to note that the degradation experiment was carried out in the same experimental conditions but devoid of our study organism, i.e., *L. stagnalis*. Therefore, the role of *L. stagnalis* and the microbiome associated with them in the uptake and degradation of both the antidepressants cannot be ruled out. Furthermore, the potential for both fluoxetine and venlafaxine to adsorb to the walls of the experimental arena (material: polystyrene) cannot be disregarded. The study by Bouly et al. (2022) reported degradation of diclofenac to metabolites in *L. stagnalis.* Gust et al. (2013) reported a higher bioaccumulation of fluoxetine in freshwater snails with bioconcentration factor (BCF) ranging between 288 and 411 in *Potamopyrgus antipodarum* (New Zealand mud snail) and between 118 and 175 in *Valvata piscinalis* (European valve snail) confirming our speculation about the uptake of fluoxetine by freshwater snails. Gomez et al. (2021) reported the BCF of venlafaxine in *Mytilus galloprovincialis* (marine mussels) to reach 265 mL/g dry weight over a 7-day exposure leading to the biological half-life of 24 ± 2 h. However, the biological half-life of both fluoxetine and venlafaxine in the presence of *L.stagnalis* is unexplored.

### Inter- and intra-individual differences

The results of our experiment displayed a large variation in the responses of the control; to account for this, a higher number of controls were used to assess the range of variation in mean velocity and cumulative duration of movement. The high variation between- and within- individuals observed in control treatment confirmed the presence of inter-and intra-individual variation in the response *of L. stagnalis* in our study. The intra- and inter-individual variation in our study is a common scenario of many other studies especially when the aim is to investigate the changes in behavior in invertebrates (Guscelli et al. 2019; Szabó et al. 2021) and mammals (Hertel et al. 2021). The standard OECD tests such as test number 243—*Lymnaea stagnalis* reproduction test—advise on the shell-length, age, parasite-free, and exhibit low mortality and be able to reproduce all-year around (OECD 2016). However, observations on inter- and intra-individual variation while determining the effects of contaminants can be informative as reported by Szabó et al. (2021) where they highlighted the variance being informative in predicting pesticide toxicity and more than just being an additional information.

## Conclusions and future steps

To the best of our understanding, we for the first time tested the effect of two antidepressants (fluoxetine and venlafaxine) with a similar mode of action on the behavior of the freshwater pond snail, *L. stagnalis*, individually, including environmentally relevant concentrations (0.01 to 50 µg/L). Additionally, we examined their effects in the presence of *Cyprinus carpio* kairomone, i.e., 5α-CPS, at the same concentration gradient (0.01 to 50 µg/L) to further explore their combined impact. Our results reveal distinct patterns of locomotion in response to these antidepressants. Venlafaxine displayed a non-monotonic dose–response relationship, stimulating movement at lower concentrations (0.01 µg/L, 0.1 µg/L, and 1 µg/L) while inhibiting it at higher concentrations (50 µg/L). In contrast, fluoxetine did not exhibit a clear dose-dependent trend and showed response values similar to the control group. Comparison of LRRs between the fluoxetine and venlafaxine experiment also showed significantly different responses for lower and environmentally concentrations (0.01 µg/L, 0.1 µg/L, and 1 µg/L), with an increase in cumulative duration of movement relative to the control as compared to a decrease in locomotion for fluoxetine. The long concentration gradient in our study design which ranged from environmentally relevant low concentrations to higher concentrations that would only be present under more extreme conditions such as wastewater spills allowed us to detect these nonlinear responses, which would not have not been possible with other study designs. Other studies often focus on higher concentrations of contaminants (exceeding environmentally relevant concentrations) and use different behavioral endpoints such as feeding behavior and seeking shelter (Godoy et al. 2020; OECD 2016). In addition, using the language of evidence aided us with a more nuanced discussion of the behavioral responses of *L. stagnalis* to the two antidepressants, as opposed to a mere rejection or acceptance of our null-hypotheses.

In our study, we found that the presence of a kairomone, 5α-CPS, reduced the intensity of effects caused by venlafaxine and fluoxetine on individual exposure. We used bile extract containing 5α-CPS, and we speculate that an interaction between the bile salts and antidepressants reduced their availability for *Lymnaea stagnalis*. Our results also highlighted the inter- and intra-individual variations in behavior of *L.stagnalis*, an important factor which requires attention in environmental risk assessments of contaminants. Variations are inevitable in the environment, and this is especially true while looking at behavioral responses. The personality of an organism has a large role in determining the way an individual organism responds to an external stimulus such as antidepressant (in the present study). As also highlighted by Nikinmaa and Anttila (2019) and Szabó et al. (2021), variance in the individual responses could be informative in predicting the risk of environmental contaminants.

In the context of this study, it is important to acknowledge that our experiments were conducted under controlled laboratory conditions. Therefore, it is necessary to conduct further research to fully understand the ecological implications of these findings in natural environments where multiple stressors coexist. Our findings, however, provide valuable insights for conducting more accurate and informed ecological risk assessments of pharmaceuticals in aquatic environments, contributing to the protection of aquatic ecosystems. Importantly, our study highlights the limitation of grouping pharmaceutical compounds together for risk assessment purposes.

### Supplementary Information

Below is the link to the electronic supplementary material.

Supplementary file1 (DOCX 53 KB)

## Data Availability

We made all raw data and R scripts used to process this data publicly available on zenodo: 10.5281/zenodo.10476932.
