# Supplementary material for: Understanding the differential impacts of two antidepressants on locomotion of freshwater snails (Lymnaea stagnalis)
Source: Environ Sci Pollut Res Int. 2024 Jan 17;31(8):12406–21. doi: 10.1007/s11356-024-31914-0 (PMC10869440; doi:10.1007/s11356-024-31914-0)
Supplement: Supplementary file 1 — Supplementary file1 (DOCX 53 KB) [file 11356_2024_31914_MOESM1_ESM.docx]

**Supplementary file**

**Understanding the differential impacts of two antidepressants on locomotion of freshwater snails (*Lymnaea stagnalis*)**

*Nandini Vasantha Raman^1^, Asmita Dubey^1,4*^, Ellen van Donk^1,2^, Eric von Elert^3^, Miquel Lürling^4^, Tânia V. Fernandes^1^, Lisette N. de Senerpont Domis^1,4,5^*

Affiliations:

*^1^*Department of Aquatic Ecology, Netherlands Institute of Ecology (NIOO-KNAW), Droevendaalsesteeg 10, 6708 PB Wageningen, The Netherlands

*^2^* Department of Environmental Biology, University of Utrecht, Utrecht, Netherlands

*^3^*Aquatic Chemical Ecology, Biocenter, Institute of Zoology, University of Cologne, Köln, Germany

^4^Department of Aquatic Ecology and Water Quality Management, Wageningen University & Research, P.O. Box 47, 6708 PB Wageningen, the Netherlands

^5^Department of Pervasive Systems, EEMCS, University of Twente & Department of Water Resources, ITC, University of Twente, the Netherlands

* Corresponding Author:

Asmita Dubey

[A.Dubey@nioo.knaw.nl](mailto:A.Dubey@nioo.knaw.nl)

#### **Results**

#### **Chemical analysis and limit of quantification**

We analyzed both stock solutions (1000 μg/L and 100 μg/L) used in the experiment and the experimental matrix by LC-MS/MS to confirm the presence of both fluoxetine and venlafaxine during the exposure period. Lower exposure concentrations in our behavioral tracking experiment could not be quantified by LC/MS-MS due to its analytical limitations. In treatments exposed to fluoxetine, quantification was possible at concentration > 0.5 μg/L. However, exposure concentration in the experimental treatments with venlafaxine < 10 μg/L was not quantifiable due to the high limit of quantification (LOQ) i.e. 0.665 μg/L.

#### **Degradation of fluoxetine and venlafaxine**

We observed a difference in degradation pattern of fluoxetine and venlafaxine in our experimental matrix under same experimental light and temperature conditions across all tested concentrations (i.e. 0, 1, 10, and 100 μg/L) over 48 h. Due to the limit of quantification for both fluoxetine and venlafaxine, concentration changes over time could not be monitored in treatments with initial concentration of 1 μg/L. In our study, we observed the initial concentration of fluoxetine to affect the DT_50_ determined. When initial concentration was 10 μg/L the DT_50_ was 7.36 h, while with 100 μg/L it was determined to be 72.4 h.

We observed minimal photodegradation of venlafaxine under our experimental conditions. Similar to fluoxetine, venlafaxine degradation kinetics was possible only in concentrations > 10 μg/L due to the high limit of quantification. Initial concentration of venlafaxine affected the DT_50_ determined i.e. 3.9 x10^7^ h for 10 μg/L and 6.2 x10^7^ h for 100 μg/L.

#### **1.3 Additional testing for blocking effect**

Upon observing the variation between the responses of the control treatments over different treatments, we tested for additional blocking effects in our experimental design in our study. We performed additional trails where the *L. stagnalis* were exposed to control treatments (no-antidepressant, no-kairomone). Figure S1 illustrates the inter-individual variation in the mean velocity (mm/s) covered by control treatments over 12 different days. We observed that mean velocity (mm/s) covered by individuals varied over days and also within a day therefore confirming the inter individual variation in our experimental design. This difference is also more prominent between the batch run in 2021 in comparison to 2022.


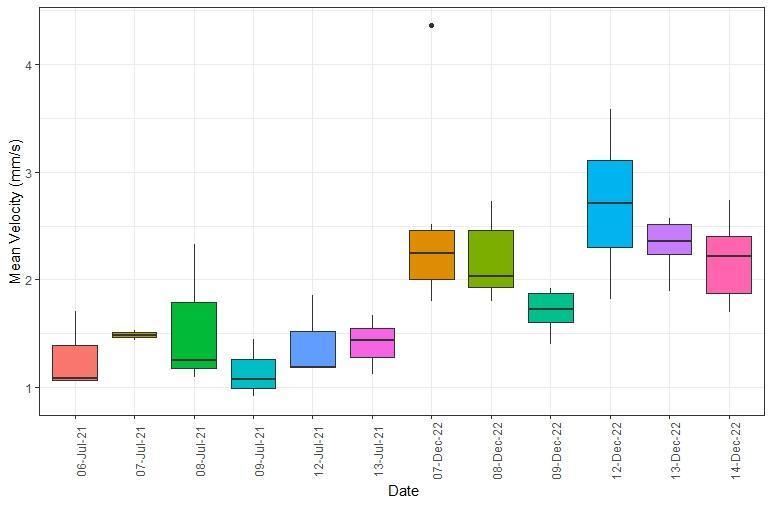


**Figure S1:** Comparison of mean velocity (mm/s) of control trails run over 12 days i, e, six trials in 2021 (6 to 9 July 2021, 12 and 13th July 2021) and six trails in 2022 (7 to 9 December 2022 and 12 to 14 December 2022).

#### **Effect size**

We calculated Cohen’s d using (Brysbaert and Stevens ,2018) to evaluate the strength of our statistical claims. We found small to large effect sizes (0.3-0.8) for the different concentrations (see Table S1).  For concentrations with lower effect size (Cohen’s d <0.3) and thus power, increase in replicates could potentially show effects that we do not see in our study.

|  | Estimate | Std.Error | df | t value | d(effect size) |
| --- | --- | --- | --- | --- | --- |
| (Intercept) | 1.27 | 0.09 | 2.98 | 13.85 |  |
| Conc0.01 | 0.14 | 0.25 | 3.89 | 0.55 | 0.25 |
| Conc0.05 | -0.17 | 0.17 | 3.02 | -1.02 | -0.44 |
| Conc0.1 | 0.47 | 0.30 | 2.47 | 1.53 | 0.69 |
| Conc0.5 | 0.49 | 0.30 | 2.67 | 1.64 | 0.75 |
| Conc1 | 0.16 | 0.13 | 2.44 | 1.24 | 0.49 |
| Conc10 | 0.15 | 0.22 | 0.67 | 0.70 | 0.32 |
| Conc50 | -0.15 | 0.11 | 3.20 | -1.33 | -0.49 |

**Table S1**: Effect size calculation
